# Head Injury as a Risk Factor for Dementia and Alzheimer’s Disease: A Systematic Review and Meta-Analysis of 32 Observational Studies

**DOI:** 10.1371/journal.pone.0169650

**Published:** 2017-01-09

**Authors:** Yanjun Li, Yongming Li, Xiaotao Li, Shuang Zhang, Jincheng Zhao, Xiaofeng Zhu, Guozhong Tian

**Affiliations:** 1 College of Basic Medicine, Jiamusi University, Jiamusi, China; 2 Department of Orthopedic Surgery, First Affiliated Hospital of Jiamusi University, Jiamusi, China; 3 Mu Dan Jiang Medical University, Mudanjiang, China; University of Arizona, UNITED STATES

## Abstract

**Background:**

Head injury is reported to be associated with increased risks of dementia and Alzheimer’s disease (AD) in many but not all the epidemiological studies. We conducted a systematic review and meta-analysis to estimate the relative effect of head injury on dementia and AD risks.

**Methods:**

Relevant cohort and case-control studies published between Jan 1, 1990, and Mar 31, 2015 were searched in PubMed, Web of Science, Scopus, and ScienceDirect. We used the random-effect model in this meta-analysis to take into account heterogeneity among studies.

**Results:**

Data from 32 studies, representing 2,013,197 individuals, 13,866 dementia events and 8,166 AD events, were included in the analysis. Overall, the pooled relative risk (RR) estimates showed that head injury significantly increased the risks of any dementia (RR = 1.63, 95% CI 1.34–1.99) and AD (RR = 1.51, 95% CI 1.26–1.80), with no evidence of publication bias. However, when considering the status of unconsciousness, head injury with loss of consciousness did not show significant association with dementia (RR = 0.92, 95% CI 0.67–1.27) and AD (RR = 1.49, 95% CI 0.91–2.43). Additionally, this positive association did not reach statistical significance in female participants.

**Conclusions:**

The findings from this meta-analysis indicate that head injury is associated with increased risks of dementia and AD.

## Introduction

The estimated annual global incidence of head injury requiring medical attention or resulting in hospitalization or death is over 10 million, and the risk of subsequent morbidity, mortality and disability is high[1].Head injury is also the major cause of loss of years of productive life and is a social problem to which governments do not pay sufficient attention[2].Neurodegenerative diseases, including amyotrophic lateral sclerosis, Parkinson’s disease and Alzheimer’s disease (AD), have frequently been reported to develop in patients with head injury[3,4]. AD is the most common neurodegenerative disorder of modern societies accounting for 50–60% of all-cause dementia[5].The possibility that head injury may predispose a person to developing AD has significant social and medical implications.

An association between head injury and AD is biologically plausible. Head injury can cause over-expression of the β-amyloid precursor protein, leading to the accumulation of β-amyloid deposits in the brain, similar to that seen in brains of AD patients[6]. Franzblau *et al*.[7]also suggest that the pathological link between head injury and AD may be due to the vascular damage, in that people with history of head injury are predisposed to AD symptoms due to altered brain vasculature; vice versa, the progression of AD pathology may be accelerated by head injury especially when the brain insult worsens hippocampal degeneration. However, the association between head injury and the risk of dementia has been debated in the epidemiological studies. Although some reports supported a positive relation with AD[8–10], other studies could not confirm head injury as a risk factor for dementia or AD[11–13].Therefore, there is no conclusive evidence to suggest a relationship between head injury and the risk of dementia.

Given the inconsistency in the literature on the role of head injury and risk of dementia and AD, we conducted a meta-analysis to quantitatively assess the relation and the strength between head injury and the risk of dementia and AD.

## Materials and Methods

### Search strategy and selection criteria

We followed the guidelines published by the Meta-analysis of Observational Studies in Epidemiology (MOOSE) group (S1 Table)[14] and the Preferred Reporting Items for Systematic Reviews and Meta-analyses (PRISMA) group (S1 Appendix) to complete the meta-analysis.We systematically searched PubMed, Web of Science, Scopus, and ScienceDirect for reports published between Jan 1, 1990, and Mar 31, 2015, using a combined text and MeSH heading search strategy with the terms: “head injuries”, “head injury”, “brain injuries”, “brain injury”, “head trauma”, “brain trauma”, “traumatic brain injury”, “brain damage”, “dementia”, “Alzheimer's disease”, “Alzheimer disease”, “AD”, “Alzheimer's”, “cognitive decline”, and “neurocognitive impairment”. We also checked the reference lists of identified reports for other potentially relevant studies. We included studies after 1990, since we sought to examine evidence from the period most applicable to the present status of dementia and AD risks associated with head injury. The search strategy was limited to cohort studies and case-control studies. No attempt was made to find articles in languages other than English. We contacted the authors of the included studies to ask them for additional information and unpublished data as needed.

A study was eligible for inclusion if the following criteria were met: (1) examination of head injury or traumatic brain injury (TBI) as the variable of interest; (2) determination of incidence of AD or other types of dementias as the outcome of interest; and (3) reporting the RRs or odds ratios (ORs) of dementia or AD with their 95% confidence intervals (CIs). The studies about animal experiment, mechanistic research and review research were excluded.

### Data extraction and study quality evaluation

We extracted the characteristics of each included study, including author, study region, study design, sample size, mean age of the sample, exposure ascertainment, exposure variable, outcome (any dementia or AD), disease ascertainment, RRs or ORs with CIs, and factors adjusted for. The most adjusted estimate was included when a study reported more than one risk estimate, and crude risk estimate was included if a study did not adjust for other factors. For the purpose of sensitivity analysis, we also extracted information on minimally adjusted (crude or adjusted for sex and age) risk estimates from each study when available. The quality of each study was assessed by the Newcastle-Ottawa Scale recommended by Wells and colleagues[15].

### Statistical analysis

A previous study indicated that OR is close to RR when the outcome is relatively uncommon (less than 20%)[16]. Thus, in our pooled analyses, ORs were considered equivalent to RRs since the incidence of dementia and AD was uncommon among the population (obviously below 20%) in the included studies. Pooled RRs were used as summary estimates throughout the procedure to simplify reporting[17].We used the random effects model to estimate the pooled RRs of dementia and AD associated with head injury to take into account heterogeneity among studies, since the study design and measuring time were varied across studies. The *I*-squared (*I*^2^) statistic and *Q*-statistic were used to explore the heterogeneity among studies. Large *I*^2^ (>50%) or *P*<0.10 for *Q*-statistic suggests substantial heterogeneity among studies. Subgroup analyses were performed according to the status of unconsciousness, including head injury regardless of status of unconsciousness, head injury with loss of consciousness (LOC), and head injury without LOC. We did several sensitivity analyses: mean age of the participants (≥65 year *vs*<65 years), sex, geographic location (Europe, North America, or Asia & Pacific), study design (cohort or case-control), exposure type (TBI or other types of head injury), disease ascertainment methods, study quality score (full marks or not full marks), and year of publication (pre-2000 or 2000 onwards). We also performed sensitivity analyses by removing each individual study from the meta-analysis. We used funnel plots to examine the presence of publication bias (ie, by plotting the natural log of the odds ratio against its standard error). Egger’s regression test and Begg-Mazumdar test were used to further assess publication bias. All statistical analyses were done with Stata Version 12.0 software (Stata Corp, College Station, TX).

## Results

### Study characteristics

**Fig 1:**
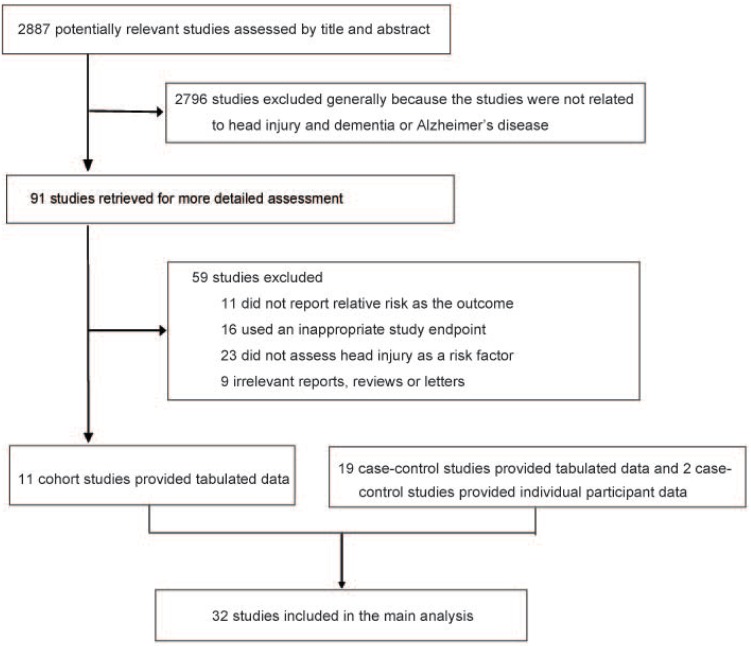
Flowchart for the selection of eligible studies.

The systematic search identified 2887 articles, which were assessed by title and abstract. Of these, 91 articles were qualified for selection (Fig 1, S2 Appendix). After full-text assessment, a total of 32 articles met the inclusion criteria and were included in the meta-analysis, including 11 cohort studies[10,12,18–26] and 21 case-control studies[8,9,11,13,27–43]. Table 1 shows the baseline characteristics of all 32 included studies. Of these studies, eleven reported on dementia incidence and twenty-eight on AD incidence. The sum number of individual studies was more than 32 because some studies reported both dementia and AD outcomes. Overall, data were available from 2,013,197 individuals, of whom 13,866 developed dementia and 8,166 developed AD. Head injury/TBI ascertainment was mainly based on detailed/structured interview with multiple questions or International Classification of Diseases codes. Thus, possible occurrence of memory bias may exist but was considered limited. The quality assessment of the included studies was presented in detail in the supplementary material (S2 and S3 Tables).

**Table 1:** Characteristics of included studies.

Author	Region	Study design	Sample size	Age, mean/range (years)	Exposure ascertainment	Exposure variable	Outcome	Disease ascertainment	RR (95% CI)	Adjustment
Abner*et al*, 2014	The United States	Cohort	649	72.9/≥60	Single question	Head injury	AD	CERAD	1.47(1.03–2.09)*	APOE-ε4, sex, age at death, presence of at least mild cerebral amyloid angiopathy, and whether AD was observed before death
									1.18(0.83–1.68)**	
Bachman*et al*, 2003	The United States	Case-control	2779	70.6/≥50	Detailed interview	Head trauma	AD	NINCDS-ADRDA	2.40(1.80–3.10)	Age, sex, education, head trauma, alcohol, and smoking
Boston *et al*, 1999	The United Kingdom	Case-control	396	82.9/≥75	Not reported	Head injury	Dementia	MMSE and CAMDEX	0.49(0.14–1.75)	Age, social class, age of left school, family history of dementia, history of falls, history of heart attack, history of hypertension, blood pressure, smoking, drinking, psychiatric history, cholesterol, and HDL
							AD		0.80(0.44–1.43)	
Broe*et al*, 1990	Australia	Case-control	340	78.1/52-96	Detailed interview	Any head injury	AD	NINCDS-ADRDA	1.33(0.46–3.83)	Age and sex
									1.75(0.52–5.88)*	
						Early head injury	AD		1.60(0.53–4.84)	
									2.33(0.63–8.67)*	
Dams-O'Connor *et al*, 2013	The United States	Cohort	4225	74.9/≥65	Single question	TBI with LOC	Dementia	DSM-IV	0.87(0.60–1.27)	Age, age-squared, gender, and education
							AD	NINCDS-ADRDA	0.95(0.65–1.38)	
Ferini-Strambi*et al*, 1990	Italy	Case-control	189	58.9/Not reported	Structured interview	Head injury	AD	Full-scale WAIS IQ and Blessed-Tomlinson-Roth Dementia Scale	1.00(0.32–3.10)	Age, sex, residential area, education and social status
Fischer *et al*, 2008	Austria	Cohort	479	75.8/75-76	Structured interview	Head trauma	AD	NINCDS-ADRDA	0.46(0.16–1.31)	None
Forster *et al*, 1995	England	Case-control	218	Not reported/<65	Standardised interview	Any head injury	AD	NINCDS-ADRDA	1.20(0.57–2.56)	Age and sex
						Adult head injury			1.50(0.68–3.41)	
						Childhoodhead injury			0.70(0.14–2.81)	
Fratiglioni*et al*, 1993	Sweden	Case-control	314	Not reported/≥75	Structured interview	TBI with LOC	AD	DSM-III-R and CDR	0.30(0.10–1.20)	Age, sex, education, type of informant, and alcohol consumption
Gardner *et al*, 2014	The United States	Cohort	164661	71.6/≥55	ICD-9	TBI	Dementia	ICD-9 codes for the diagnosis of dementia	1.26(1.21–1.32)	Age, sex, race, comorbidities, trauma mechanism, health care use, and trauma severity
Graves *et al*, 1990	The United States	Case-control	260	64.9/Not reported	Structured interview	Any head trauma	AD	DSM-III and NINCDS-ADRDA	3.50(1.50–8.30)	Age and family history of AD
						Head traumawith LOC			2.90(1.10–7.53)	
						Head trauma without LOC			5.50(1.35–22.50)	
Guo*et al*, 2000	The United States, Canada, and Germany	Case-control	16901	Not reported	Structured interview	Any head injury	AD	NINCDS-ADRDA	2.70(2.20–3.30)	Gender and kinship
						Head injury with LOC			4.00(2.90–5.50)	
						Head injury without LOC			2.00(1.50–2.70)	
Lee *et al*, 2013	Taiwan	Cohort	720933	Not reported/≥18	ICD-9	Mild TBI	Dementia	ICD-9-CM	3.26(2.69–3.94)	Age, gender, urbanization level, socio-economic status, diabetes, hyperlipidemia coronary artery disease, history of alcohol intoxication, ischemic stroke, intracranial hemorrhage and Charlson comorbidity index
Li *et al*, 1992	China	Case-control	210	65.3/Not reported	Structured interview	Head injury with LOC	AD	NINCDS-ADRDA and ICD-10	1.00(0.09–11.03)	Age and sex
Lindsay *et al*, 2002	Canada	Case-control	4088	73.3/≥65	Structured interview	Head injury	AD	NINCDS-ADRDA	0.87(0.56–1.36)	Age, sex and education
Luukinen*et al*, 2005	Finland	Cohort	152	75.1/≥70	Medical examination	TBI	Dementia	DSM-IV and the MMSE	2.80(1.35–5.81)	Low educational status and sex
Mayeux*et al*, 1993	The United States	Case-control	331	78.1/Not reported	Structured interview	Head injury with LOC	AD	National Institutes of Neurological Disorders and Stroke criteria	3.70(1.40–9.70)	Gender, age, ethnic group, years of education, and head injury
McDowell *et al*, 1994	Canada	Case-control	793	80.9/≥65	Structured interview	Head injury	AD	DSM-III-R and NINCDS-ADRDA	1.66(0.97–2.84)	Age, sex, residence in community or institution, and education
Mehta *et al*, 1999	The Netherlands	Cohort	6645	68.9/≥55	Detailed interview	Head trauma with LOC	Dementia	DSM-III-R and NINCDS-ADRDA	1.00(0.50–2.00)	Age, education, and if applicable, gender
							AD		0.80(0.40–1.90)	
							Dementia		0.70(0.20–2.40)*	
							AD		0.90(0.20–4.00)*	
							Dementia		1.30(0.60–2.80)**	
							AD		0.90(0.30–2.40)**	
Nordstrom *et al*, 2014	Sweden	Cohort	811622	18.0/Not reported	ICD-8,9,10	One mild TBI	Dementia	ICD-8, 9, 10	1.50(1.10–2.00)*	Age, place and year of conscription, overall cognitive function, alcohol intoxication, weight, height, knee extension strength, TBI in parents, dementia in parents, income, educational level, systolic blood pressure, drug intoxication, depression, and cerebrovascular disease
							AD		1.00(0.50–2.00)*	
						At least two mild TBI	Dementia		1.80(1.10–3.00)*	
							AD		2.50(0.80–8.10)*	
						One severe TBI	Dementia		2.30(1.50–3.60)*	
							AD		0.70(0.10–5.20)*	
O'Meara *et al*, 1997	The United States	Case-control	691	78.0/≥60	Detailed interview	Head injury with LOC	AD	DSM-III-R and NINCDS-ADRDA	2.10(1.10–3.80)	None
									4.20(1.50–11.50)*	
									1.10(0.50–2.60)**	
Ogunniyi*et al*, 2006	The United States	Case-control	523	77.9/Not reported	Screening interview	Head injury	AD	NINCDS-ADRDA	0.55(0.21–1.45))	Age and gender
	Nigeria		470	79.2/Not reported					1.36(0.30–6.30))	
Plassman*et al*, 2000	The United States	Cohort	1776	72.9/Not reported	Medical record	Head injury	Dementia	DSM-III-R and NINCDS-ADRDA	2.46(1.43–4.24)*	Years of education and age
							AD		2.16(1.10–4.23)*	
Rasmusson*et al*, 1995	The United States	Case-control	102	71.0/Not reported	Standardized interview	Head injury	AD	Not reported	13.75(1.76–107.52)	None
Rippon *et al*, 2006	The United States	Case-control	1498	68.2/Not-reported	Standardized interview	Head injury	AD	NINCDS-ADRDA	1.00(0.70–1.60)	ε4 status, age, gender, and education
Salib*et al*, 1997	The United Kingdom	Case-control	538	75.1/>65		Head injury	DementiaAD	NINCDS-ADRDA	2.46(1.42–4.10)	Age, sex, time lag between head injury and onset, duration of condition and family history of dementia
									1.52(0.98–2.35)	
									2.10(1.10–4.10)*	
									1.38(0.74–2.60)**	
Schofield *et al*, 1997	The United States	Cohort	271	75.3/≥60	Detailed interview	Head injury with LOC	AD	NINCDS-ADRDA	1.05(0.34–3.22)	Sex and education
Suhanov*et al*, 2006	Russia	Case-control	520	69.3/40-89	Structured interview	Head injury with LOC	AD	NINCDS-ADRDA	1.70(1.00–2.80)	Family history of dementia, family history of parkinsonism, and hypertension
Sundstrom*et al*, 2007	Sweden	Case-control	543	72.8/40-85	Medical record or interview	Mild head injury	Dementia	DSM-IV	0.90(0.40–1.80)	Age and gender
Tsolaki*et al*, 1997	Greece	Case-control	134	Not reported/>70	Structured interview	Head trauma	AD	DSM-IV and NINCDS-ADRDA	1.07(0.47–2.45)	Age and gender
vanDuijn*et al*, 1992	The Netherlands	Case-control	396	56.8/Not reported	Structured interview	Head trauma with LOC	AD	NINCDS-ADRDA	1.60(0.80–3.40)	Age, sex, dementia in first-degree relatives and education
									2.50(0.90–7.00)*	
									0.90(0.30–2.80)**	
Wang *et al*, 2012	Taiwan	Cohort	269550	40.8/≥15	ICD-9	TBI	Vascular dementia	ICD-9-CM	1.32(1.06–1.65)	Sex, age group, year of index healthcare use, stroke, diabetes, hyperlipidaemia, hypertension, coronary heart disease, heart failure and arterial fibrillation
							Unspecific dementia		1.74(1.62–1.88)	
							AD		1.49(1.08–2.07)	

RR = relative risk. CI = confidence interval. AD = Alzheimer’s disease. CERAD = The Consortium to Establish a Registry for Alzheimer's Disease. MMSE = Mini-Mental State Examination test. CAMDEX = Cambridge Examination for Mental Disorders of the Elderly. TBI = traumatic brain injury. LOC = loss of consciousness. DSM-IV = Diagnostic and Statistical Manual of Mental Disorders, Fourth Edition. NINCDS-ADRDA = National Institute of Neurological and Communicative Diseases and Stroke-Alzheimer Disease and Related Disorders Association. DSM-III-R = Diagnostic and Statistical Manual of Mental Disorders, third edition revised. ICD-9 = International Classification of Diseases, Ninth Revision. CDR = The Clinical Dementia Rating scale.

* Relative risks and 95% CIs for males.

** Relative risks and 95% CIs for females.

### Head injury and risk of dementia

**Fig 2:**
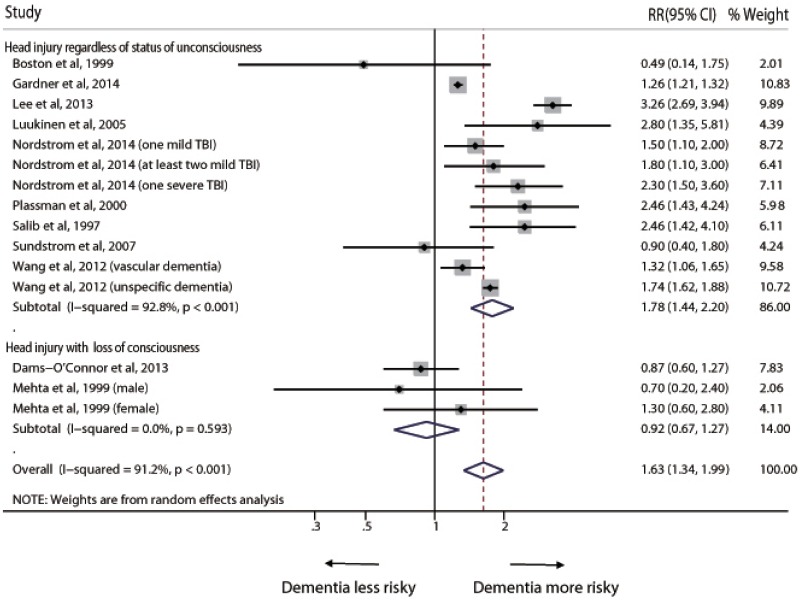
Pooled relative risk for any dementia, comparing individuals with head injury to those without head injury. Box sizes are in proportion to study weights. TBI = traumatic brain injury.

The overall pooled RR based on all available data for any dementia risk associated with head injury was 1.63 (95% CI 1.34–1.99) (Fig 2). The *I*^*2*^ statistic for heterogeneity between studies was 91.2%, with p value for the *Q* test <0.001, suggesting substantial between-study heterogeneity. In the sub-group analyses, when regardless of status of unconsciousness, the pooled RR was 1.78 (95% CI 1.44–2.20); however, head injury with LOC did not show significant association with risk of dementia (RR = 0.92, 95% CI 0.67–1.27) (Fig 2).

### Head injury and risk of AD

**Fig 3:**
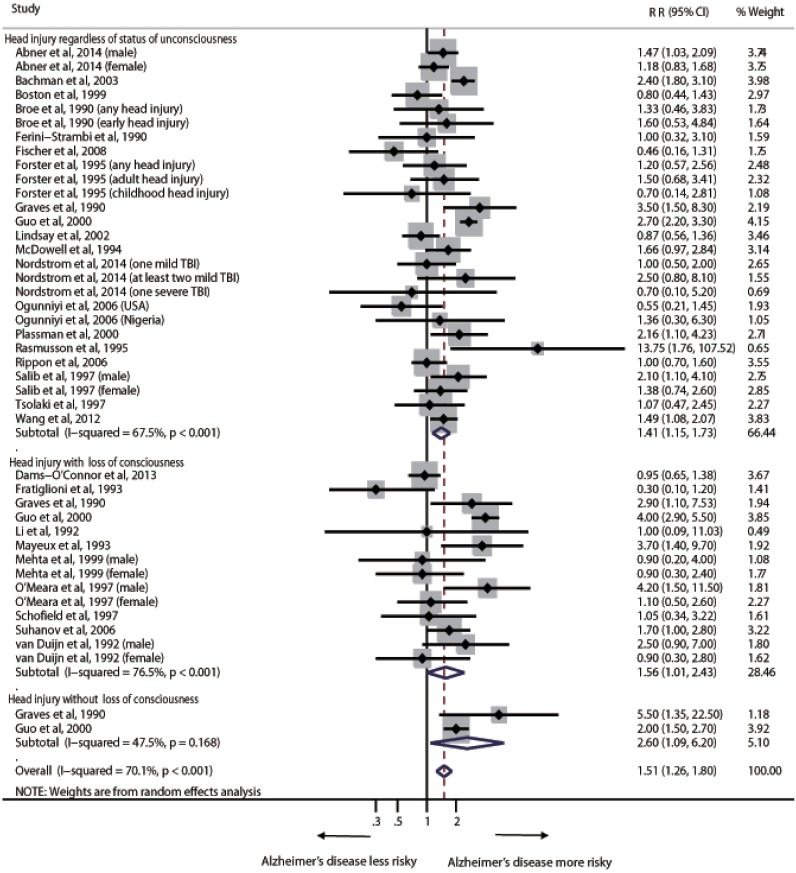
Pooled relative risk for Alzheimer’s disease, comparing individuals with head injury to those without head injury. Box sizes are in proportion to study weights. TBI = traumatic brain injury.

The pooled RR estimates for risk of AD associated with head injury was 1.51(95% CI 1.26–1.80) (Fig 3). The *I*^*2*^ statistic for heterogeneity between studies was 70.1%, with p value for the *Q* test <0.001, suggesting substantial between-study heterogeneity. In the subgroup analyses, head injury regardless of status of unconsciousness (RR = 1.41, 95% CI 1.15–1.73), head injury with LOC (RR = 1.56, 95% CI 1.01–2.43) and head injury without LOC (RR = 2.60, 95% CI 1.09–6.20) were all associated with increased risk of AD (Fig 3). Exclusion of the three studies with results not adjusted for other factors did not change the RR estimates (1.50 [1.26–1.80]) and did not reduce the between–study heterogeneity (*I*^*2*^ = 69.9%, p<0.001) (S1 Fig). However, the results did not show significant association between head injury with LOC and risk of AD (RR = 1.49, 95% CI 0.91–2.43) (S1 Fig).

### Sensitivity analyses

**Fig 4:**
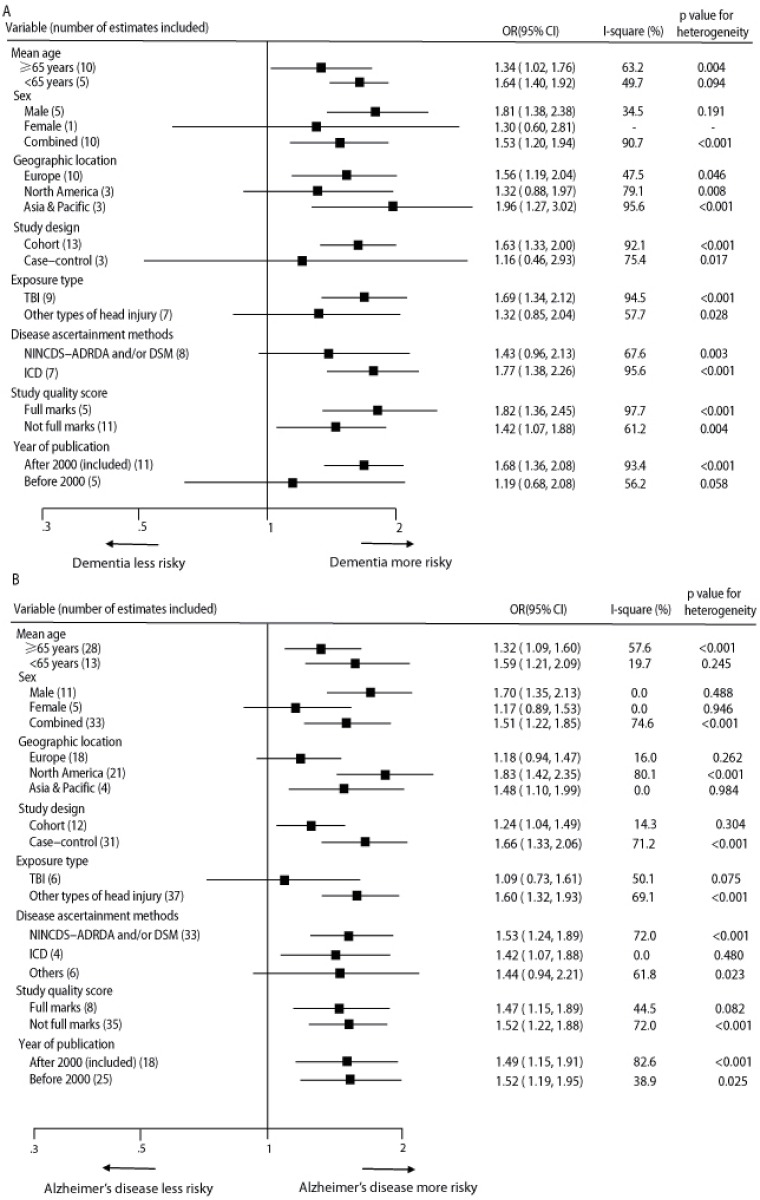
Sensitivity analyses for estimated risks of any dementia (A) and Alzheimer’s disease (B). TBI = traumatic brain injury. NINCDS-ADRDA = National Institute of Neurological and Communicative Diseases and Stroke-Alzheimer Disease and Related Disorders Association. DSM = Diagnostic and Statistical Manual of Mental Disorders.

In the sensitivity analyses, the pooled RRs for any dementia and AD did not differ significantly by mean age of the participants, sex, geographic location, study design, exposure type of head injury, disease ascertainment methods, study quality score, or year of publication (Fig 4). However, the RRs of any dementia and AD associated with head injury did not reach statistical significances in female participants, although the point estimates were over 1 (RR = 1.30, 95% CI 0.60–2.81, and RR = 1.17, 95% CI 0.89–1.53, respectively). When considering the types of head injury, TBI rather than other types of head injury showed significant association with increased risk of any dementia (RR = 1.69, 95% CI 1.34–2.12, and RR = 1.32, 95% CI 0.85–2.04, respectively), while other types of head injury rather than TBI showed significant association with increased risk of AD (RR = 1.60, 95% CI 1.32–1.93, and RR = 1.09, 95% CI 0.73–1.61, respectively). Additionally, the positive associations were not materially changed in the leave-one-out analyses by omitting one study in turn, with a pooled RR of any dementia range from 1.51 (95% CI 1.27–1.79) to 1.72 (95% CI 1.40–2.11) (S2 Fig), and a pooled RR of AD range from 1.45 (95% CI 1.23–1.72) to 1.54 (95% CI 1.29–1.84) (S3 Fig).In the sensitivity analyses, 11 risk estimates for dementia from 7 studies and 36 estimates for AD from 21 studies originally reporting minimally adjusted RRs with 95% CIs were included. The results were in line with the pooled estimates found in meta-analyses (data not shown).

### Publication bias

**Fig 5:**
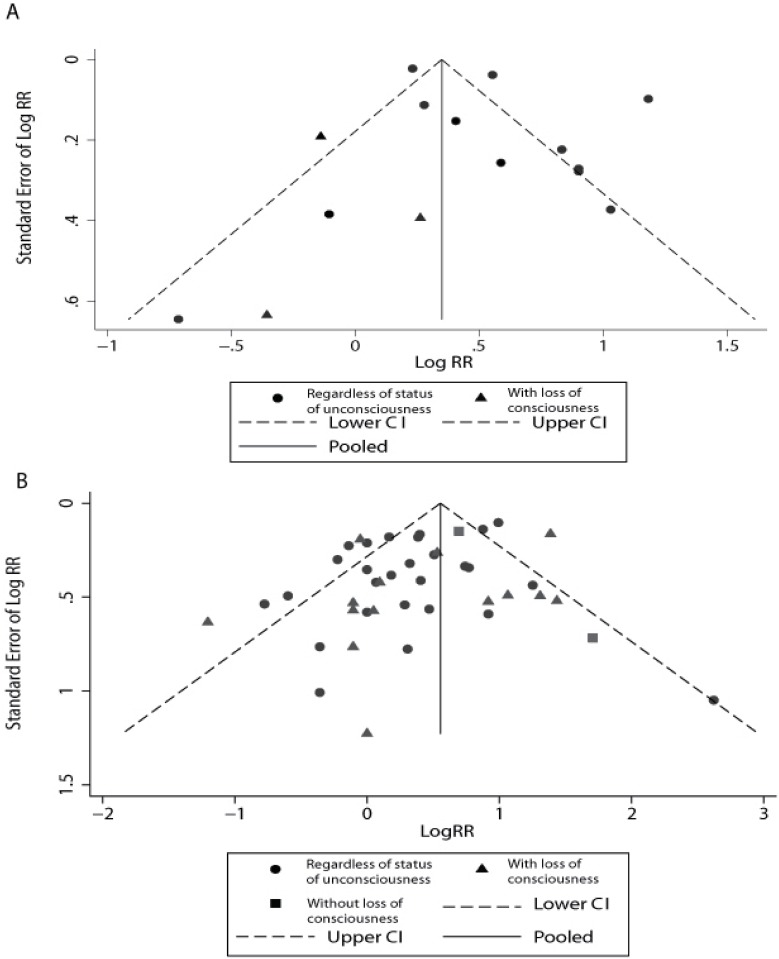
Funnel plot to explore publication bias in the estimates of any dementia (A) and Alzheimer’s disease (B). The vertical line is at the mean effect size.

Visual assessment of funnel plots showed that the studies were distributed fairly symmetrically about the combined effect size in both meta-analyses (Fig 5), which suggests little publication bias in our meta-analyses. Egger’s regression test (*P* = 0.327 and *P* = 0.139, respectively) and Begg-Mazumdar test (*P* = 0.255 and *P* = 0.958, respectively) further confirmed that there was no potential publication bias in both meta-analyses.

## Discussion

In this pooled analysis of 32 observational studies, with data for more than two million individuals and more than 13,000 dementia events and 8,000 AD events, head injury was a risk factor for any dementia and AD. Indeed, we observed an overall 63% increase in any dementia risk and 51% increase in AD, comparing individuals with head injury to those without head injury. These findings add to the accumulating evidence that head injury may lead to neurodegenerative diseases, although current evidence from the literature is mixed.

The findings of this pooled analysis are partially consistent with the positive association observed between head injury and AD observed in a meta-analysis that combined case-control studies that were conducted up to the year 2001[44]. The previous meta-analysis did not assess the relationship between head injury and risk of any dementia, and only included case-control studies that published before 2001. Our analysis included more studies than did the previous meta-analysis (11 compared with none and 28 compared with 15 for studies examining any dementia and AD, respectively), especially included many studies that published after 2001. Moreover, in our meta-analysis, we considered the status of unconsciousness and conducted sensitivity analyses for more variables in addition to gender, and the pooled estimate of the AD risk (RR = 1.51) was more precise (95% CI 1.26–1.80) than that of the other study. Perry *et al* assessed the relationship between TBI and subsequent neurological and psychiatric disease in a meta-analysis[45], using dementia and AD as subgroups of the overall analysis. Similarly, the studies relevant to dementia and AD were not enough comprehensive in their study (only included seven studies for dementia and nineteen for AD) and the relationship between TBI and dementia/AD was not well discussed. The relationship between any head injury and dementia/AD was not assessed in their meta-analysis either. In addition, Perry *et al*’s study did not find significant association between TBI and dementia (OR = 1.36, 95% CI 0.84–2.19), which is inconsistent with our result. This might be due to the limited number of studies that included in their meta-analysis.

Our meta-analysis included not only retrospective case-control studies but also prospective cohort studies. An important strength of prospective studies is that recruitment takes place and information about head injury is recorded before participants know whether they will develop dementia or AD. The robustness of prospective data is demonstrated by the stability of the findings in the sensitivity analyses. When the retrospective studies were assessed in isolation in the sensitivity analyses, their aggregate findings differed from those of the prospective studies, which may be caused by biases in some retrospective studies. Many retrospective study results could have been somewhat biased by selective participation of head injury patients, and in all retrospective studies information about head injury was recorded after dementia or AD diagnosis, so there might have been differential recall of head injury.

An interesting finding in our subgroup analyses indicated that head injury with LOC had no significant association with dementia, and had no significant association with AD in the meta-analysis that excluded studies with results not adjusted for other factors. However, head injury without LOC showed consistently positive association with AD. This finding conflicted with the preconceived opinion that more severe injury induces more serious complications. One primary possible explanation was that most included studies did not distinguish head injury with and without LOC. Thus, there were very limited studies in the head injury with LOC or without LOC subgroup, making the results of subgroup low of statistical power. More studies are needed to further assess the relative risk of dementia/AD induced by head injury with LOC and without LOC separately. Another explanation was that head injuries without LOC would be susceptible to greater recall bias, and if that were so, one might observe a greater risk for AD among head injured persons without than those with LOC[31]. Also, there may be a survivor bias, where people with history of more severe head injury who later enrolled in studies or survived into old age were the best able to recover from those injuries. In addition, the idea of the early pre clinical minor motor features of dementia leading to falls and minor head injury seems a much more probable explanation for our findings. Moreover, residual or unmeasured confounding factors, such as alcohol consumption, misuse prescribed opiates, and other psychiatric illnesses such as depression may also contribute to this anomalous result. Although this finding is consistent with the result of a large EURODEM pooled analysis of four European population-based studies which showed head trauma with unconsciousness was not associated with AD[46], the mechanism that the influence of head injury severity on dementia and AD need to be further clarified.

In the sensitivity analyses by sex, the positive association between head injury and dementia/AD did not reach statistical significance in female participants. The previous meta-analysis also indicated that the excess risk of head injury in those with AD is only found in males[44]. The sex difference in the risk of dementia and AD following head injury may contribute to the role of the female hormones, oestrogen and progesterone. Animal models of stroke and TBI have suggested that these hormones may confer a neuroprotective and neuroregenerative effect[47,48]. In the animal model conducted by Bramlett and Dietrich[49],neuropathological protection effect after TBI was found in intact female rats versus males or ovariectomized females. Their results provided evidence for endogenous hormonal histopathological protection following brain injury. Moreover, oestrogen has been reported as a protective factor in the development of AD.

The strengths of the present meta-analysis include that almost all the worldwide evidence from eligible epidemiological studies was included, as well as the acceptable methodologic quality of the studies on which the analysis is based. One limitation of our study is that all types of “head injury” were treated equally in the pooled analyses. Although we did sensitivity analyses by exposure type (TBI or other types of head injury), the results show that other types of head injury didn’t show significant association with increased risk of any dementia and TBI didn’t show significant association with increased risk of AD. This may be due to the limited number of studies included in the sensitivity analyses. More studies are needed to further clarify the relationship between a true TBI and the risk of dementia and AD. Other possible limitations of the study include the heterogeneity between the studies, including studies in which outcomes were recorded with different scales. Defining the status of head injury at baseline (cohort studies) or during the reference period (case-control studies) possibly caused the heterogeneity between the included studies, because the follow-up periods and the reference dates varied between the studies. Moreover, there were numerous differences in the analytic methods used to estimate the ORs and RRs among different studies, which may also contribute to the heterogeneity in the results. Therefore, our results must be interpreted with caution. We assumed that the true effect would vary between the studies because of potential additional heterogeneity in the populations, designs, and analyses of the various studies, in addition to the usual sampling variation in the estimates within studies. To account for the heterogeneity, we used the random effects model to combine the results of the original studies. The random-effect approach provides some allowance for heterogeneity in studies beyond sampling error. One can expect a very limited influence of heterogeneity by using the random effects model, although this does not necessarily rule out the effect of heterogeneity between the studies. Sensitivity analyses by some study-level factors were performed to further explore the sources of heterogeneity, but heterogeneity persisted after we performed relevant sensitivity analyses. Although the studies included in our meta-analysis were heterogeneous, the relation is largely consistent.

Our meta-analysis is based on observational studies, and the possibility of selection bias, misclassification bias related to exposure, and failure to consider residual or unmeasured confounding cannot be ruled out. The assessment methods for head injury may also vary between the studies. The assessment in several included studies was based on self-reported questionnaire and medical records documenting the severity of injury were not accessed for all participants, and such data are subject to recall bias, especially for the patients with dementia and AD. Moreover, the present data do not address the important issues of whether a single head injury and repeated head injury can both increase the risk of dementia and AD, and whether a recent injury and an earlier injury on head or whether mild TBI and serious TBI have different impact on the risk of dementia and AD. Unfortunately, the data available in the current study do not allow for more refined categorisations of head injury. The time elapsed between head injury and dementia symptoms starting was reported rarely among the included studies, and this may be another limitation of this study. Besides, without autopsy confirmation, clinical diagnosis of AD is suspect due to all the “AD mimics”(hippocampal sclerosis, primary age-related lalopathy, Lewy body disease, vascular dementia, etc).

Limitations aside, this meta-analysis remains the most comprehensive study to date that addressed the association between head injury and risk of dementia and AD. The diversity of location, ethnicity, age of participants, and head injury status reported in these studies also allows for increased generalisability of these results to other populations. However, studies included in our meta-analysis are mainly from Europe and The United States, thus, the results of our study must be interpreted with caution when generalized to populations from other regions. Although we recognize the methodological limitations of the studies included in this meta-analysis, our study does serve as a comprehensive review of this literature.

### Conclusion

On the basis of epidemiologic evidence, we found that head injury was associated with an increased risk of dementia and AD. For further studies, based on our findings, we suggest that the investigators should improve the standardization of various assessment methods of head injury, dementia and AD. Furthermore, this study adds to the existing evidence that head injury may lead to neurodegenerative diseases, and the use of genetic and biological makers as surrogate end points in the future studies should help to clarify the case and effect relationship that links head injury and dementia and AD.

## Supporting Information

S1 Appendix PRISMA 2009 Checklist. (DOC)

S2 Appendix PRISMA 2009 Flow Diagram. (TIF)

S1 Fig Pooled relative risk for Alzheimer’s disease after exclusion of studies with results not adjusted for multiple covariates, comparing individuals with head injury to those without head injury. Box sizes are in proportion to study weight. TBI = traumatic brain injury.
(TIF)

S2 Fig Sensitivity analyses. Pooled relative risks for any dementia associated with head injury by omitting one study in turn.
(TIF)

S3 Fig Sensitivity analyses. Pooled relative risks for Alzheimer’s disease associated with head injury by omitting one study in turn.
(TIF)

S1 Table MOOSE Checklist. (DOCX)

S2 Table Quality assessment of the included studies (cohort studies). (DOCX)

S3 Table Quality assessment of the included studies (case-control studies). (DOCX)
