# Supplementary figures and images for: Head Injury as a Risk Factor for Dementia and Alzheimer’s Disease: A Systematic Review and Meta-Analysis of 32 Observational Studies
Source: PLoS One. 2017 Jan 9;12(1):e0169650. doi: 10.1371/journal.pone.0169650 (PMC5221805; doi:10.1371/journal.pone.0169650)

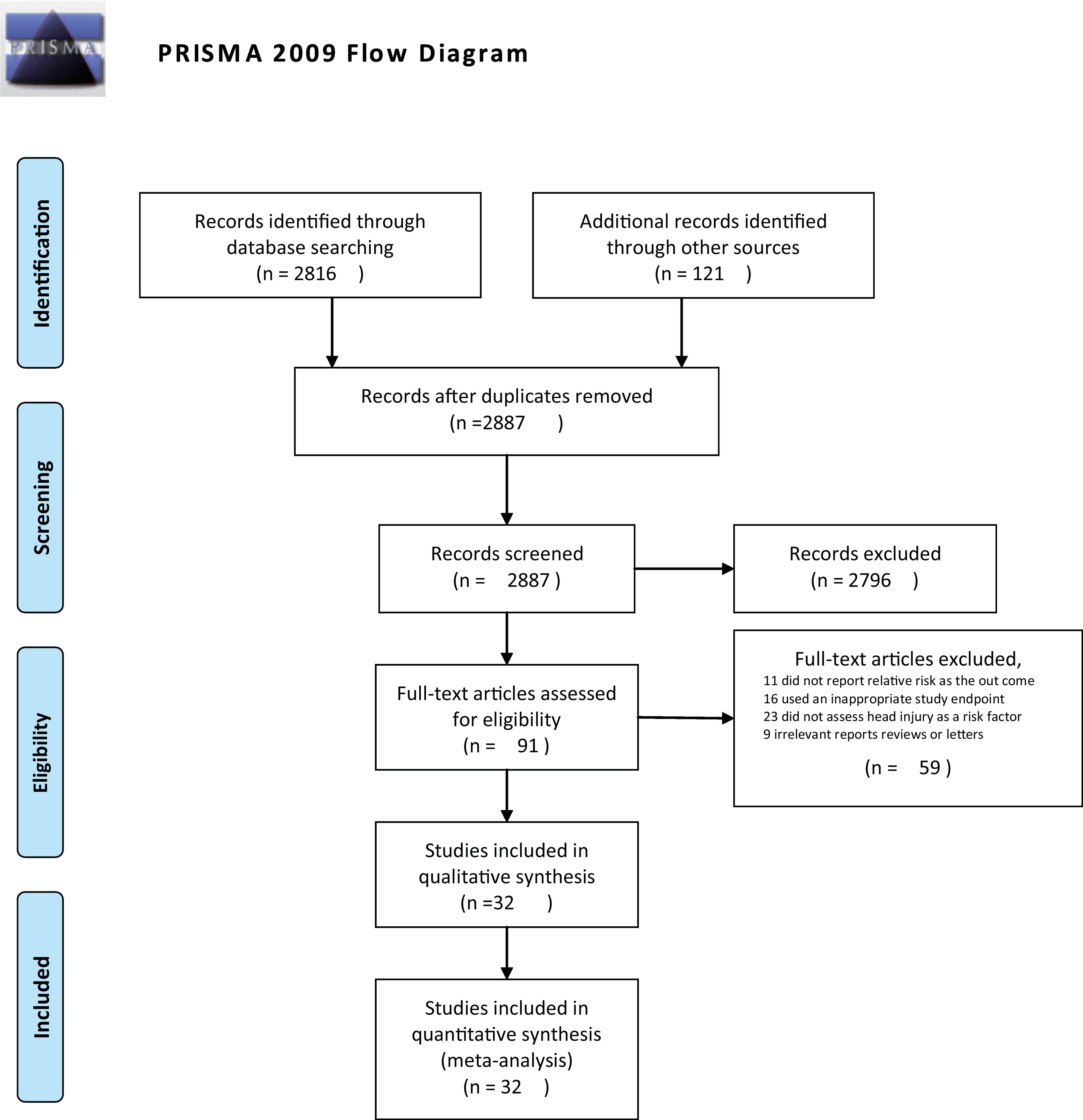

Supplement: S2 Appendix — (TIF) [file pone.0169650.s002.tif]

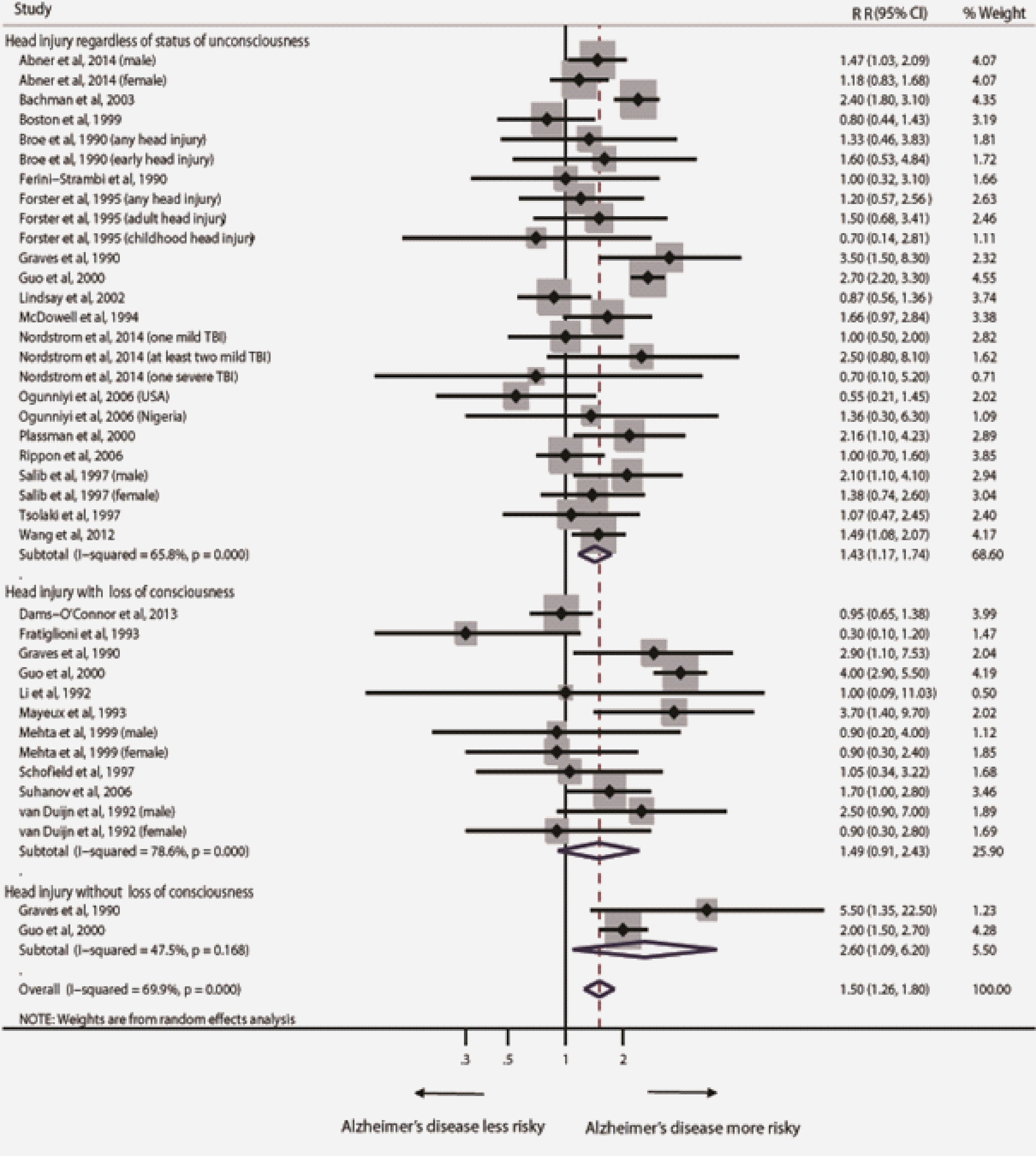

Supplement: S1 Fig — Box sizes are in proportion to study weight. TBI = traumatic brain injury. (TIF) [file pone.0169650.s003.tif]

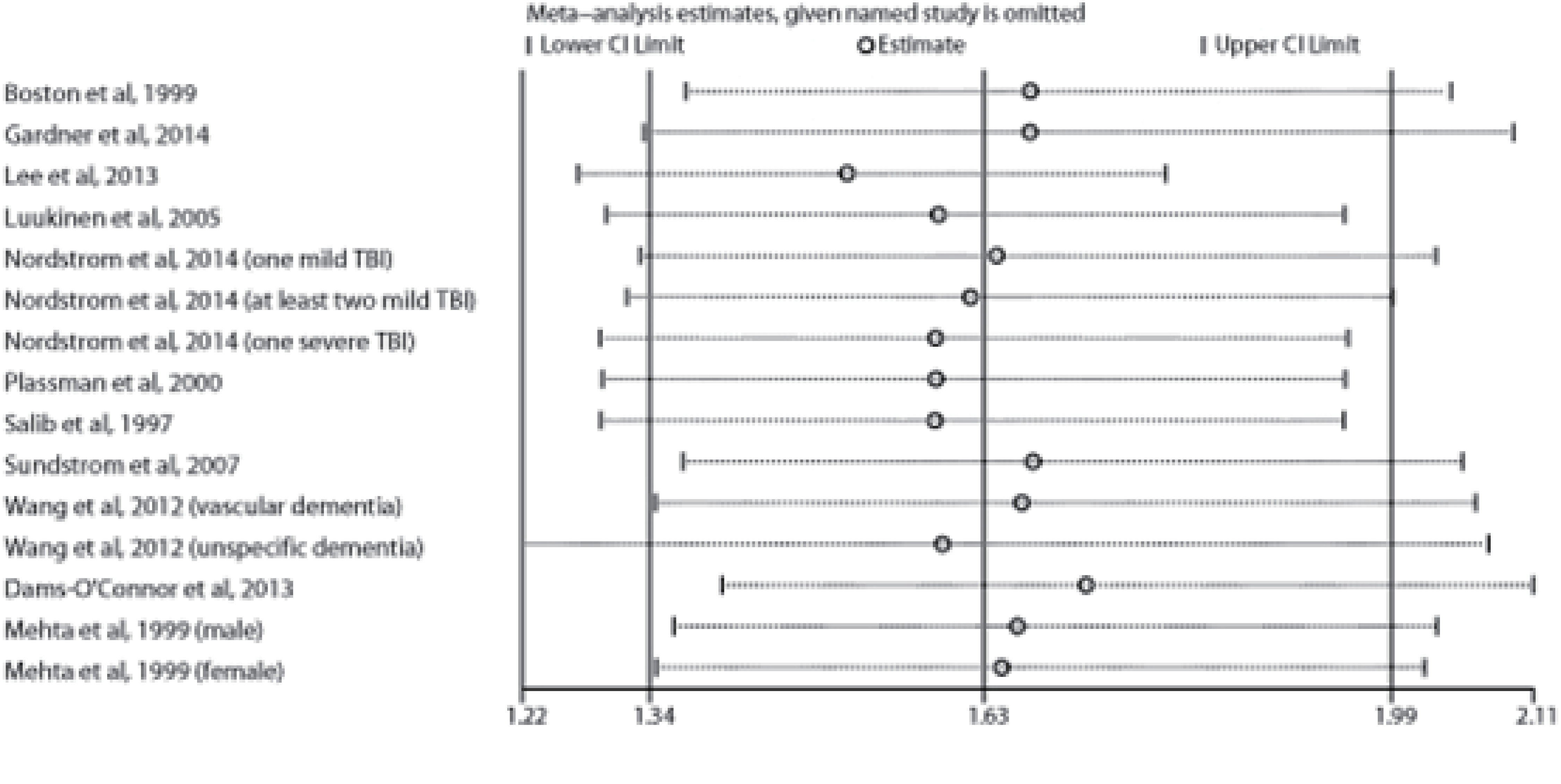

Supplement: S2 Fig — Pooled relative risks for any dementia associated with head injury by omitting one study in turn. (TIF) [file pone.0169650.s004.tif]

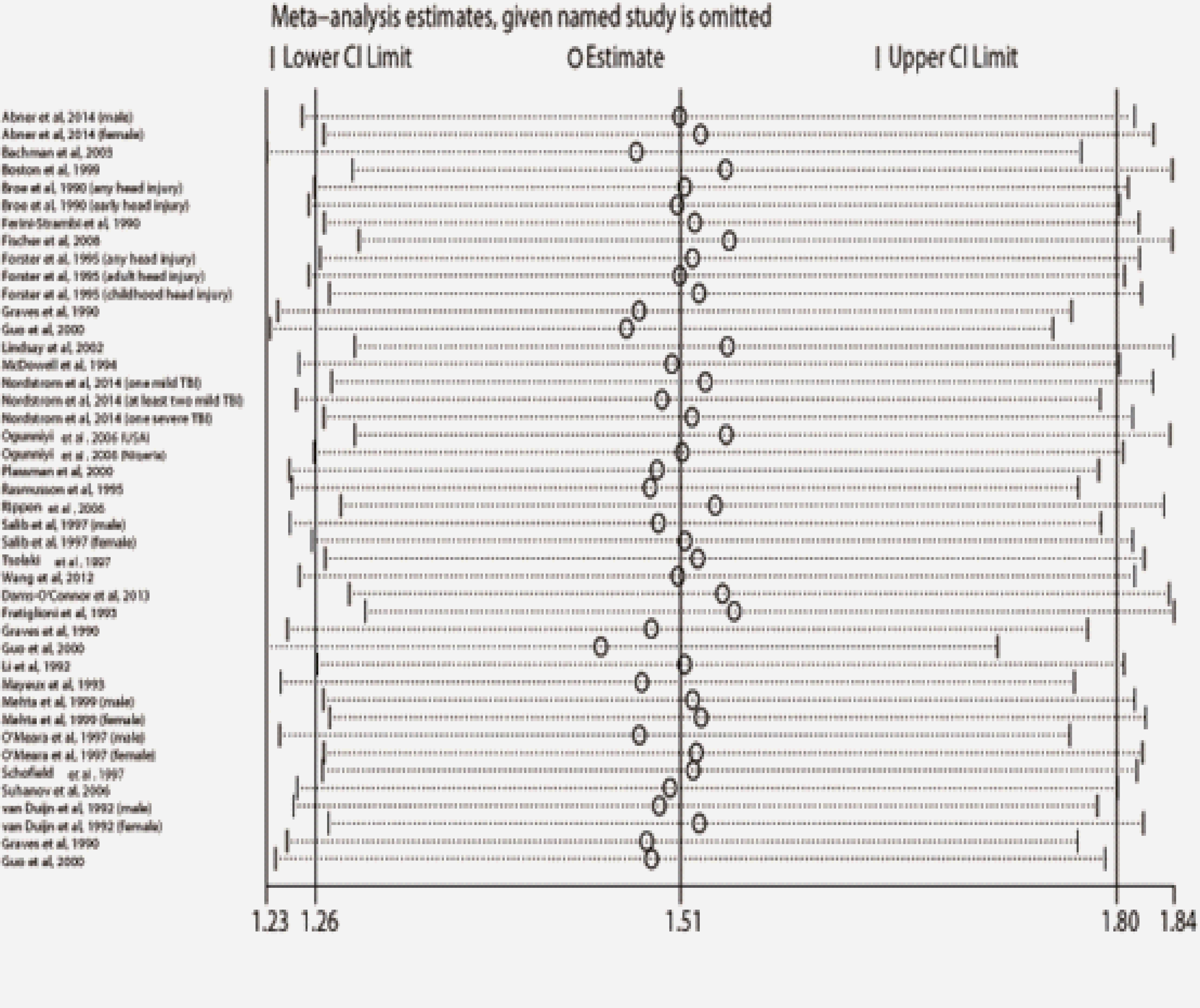

Supplement: S3 Fig — Pooled relative risks for Alzheimer’s disease associated with head injury by omitting one study in turn. (TIF) [file pone.0169650.s005.tif]
