# Supplementary material for: Head Injury as a Risk Factor for Dementia and Alzheimer’s Disease: A Systematic Review and Meta-Analysis of 32 Observational Studies
Source: PLoS One. 2017 Jan 9;12(1):e0169650. doi: 10.1371/journal.pone.0169650 (PMC5221805; doi:10.1371/journal.pone.0169650)
Supplement: S1 Table — (DOCX) [file pone.0169650.s006.docx]

**S1 Table. MOOSE Checklist**

| **Criteria** | | **Brief description of how the criteria were handled in the meta-analysis** |
| --- | --- | --- |
| **Reporting of background should include** | |  |
| √ | Problem definition | The association between head injury and the risk of dementia has been debated in the epidemiological studies, and it remains to be summarized quantitatively. |
| √ | Hypothesis statement | Head injury is associated with increased risk of dementia and Alzheimer’s disease (AD). |
| √ | Description of study outcomes | Dementia and AD. |
| √ | Type of exposure or intervention used | Head injury (with loss of consciousness and without loss of consciousness). |
| √ | Type of study designs used | Cohort studies and case-control studies. |
| √ | Study population | Participants with head injury and controls. |
| **Reporting of search strategy should include** | |  |
| √ | Qualifications of searchers (eg, librarians and investigators) | The credentials of the two investigators Y.M.L. and X.L. are provided in the author list. |
| √ | Search strategy, including time period included in the synthesis and keywords | PubMed, Web of Science, Scopus, and ScienceDirect for reports published between Jan 1, 1990, and Mar 31, 2015.  Keywords: "head injuries", "head injury", "brain injuries", "brain injury", "head trauma", "brain trauma", "traumatic brain injury", "brain damage", "dementia", "Alzheimer's disease", "Alzheimer disease", "AD", "Alzheimer's", "cognitive decline", and "neurocognitive impairment". |
| √ | Effort to include all available studies, including contact with authors | References of all retrieved articles and recent reviews were reviewed. |
| √ | Databases and registries searched | PubMed, Web of Science, Scopus, and ScienceDirect |
| √ | Search software used, name and version, including special features used (eg, explosion) | We did not employ a special search software. |
| √ | Use of hand searching (eg, reference lists of obtained articles) | References of all retrieved articles and recent reviews were reviewed. |
| √ | List of citations located and those excluded, including justification | Details of the literature search process are outlined in the flow chart. |
| √ | Method of addressing articles published in languages other than English | We placed restrictions on English. |
| √ | Method of handling abstracts and unpublished studies | The search process was not restricted upon full-text articles, but also conference abstracts and unpublished studies. |
| √ | Description of any contact with authors. | We contacted the authors of the included studies to ask them for additional information and unpublished data as needed. |
| **Reporting of methods should include** | |  |
| √ | Description of relevance or appropriateness of studies assembled for assessing the hypothesis to be tested | The inclusion criteria are presented in the “Search strategy and eligibility criteria” section. |
| √ | Rationale for the selection and coding of data (eg, sound clinical principles or convenience) | We extracted the characteristics of each included study, including author, study region, study design, sample size, mean age of the sample, exposure variable, outcome (any dementia or AD), disease ascertainment, risk estimates with CIs, and factors adjusted for. The most adjusted estimate was included when a study reported more than one risk estimate. |
| √ | Documentation of how data were classified and coded (eg, multiple raters, blinding, and inrerrater reliability) | Data were independently extracted and analyzed by two investigators (Y.M.L. and X.L.) and final decision was reached by consensus. |
| √ | Assessment of confounding (eg, comparability of cases and controls in studies where appropriate) | Table 1 presents the adjustment factors for each study. |
| √ | Assessment of study quality, including blinding of quality assessors; stratification or regression on possible predictiors of study results | The quality of each study was assessed by two investigators (Y.M.L. and X.L.), using the Newcastle-Ottawa Scale. |
| √ | Assessment of heterogeneity | The *Q*-statistic and *I*-squared (*I*^2^) statistic were used to explore the heterogeneity among studies. |
| √ | Description of statistical methods (eg, complete description of fixed or random effects models, justification of whether the chosen models account for predictors of study results, dose-response models, or cumulative meta-analysis) in sufficient detail to be replicated | Description of methods of meta-analyses, subgroup analyses, and assessment of publication bias are detailed in the “Statistical analysis” section. |
| √ | Provision of appropriate tables and graphics | One main table and three supplemental tables are provided. One flow chart and four forest plots appear in the main text. |
| **Reporting of results should include** | |  |
| √ | Graph summarizing individual study estimates and overall estimate | Figures 2-3 |
| √ | Table giving descriptive information for each study included | Table 1 |
| √ | Results of sensitivity testing (eg, subgroup analysis) | “Results” section; Figure 4 |
| √ | Indication of statistical uncertainty of findings | 95% confidence intervals were presented with all summary effect estimates. |
| **Reporting of discussion should include** | |  |
| √ | Quantitative assessment of bias (eg, publication bias) | Figure 5, “Results” section and “Discussion” section. |
| √ | Justification for exclusion (eg, exclusion of non-English-language citations) | The details of the exclusion of studies are shown in Flow chart. |
| √ | Assessment of quality of included studies | Tables S2-S3 |
| **Reporting of conclusions should include** | |  |
| √ | Consideration of alternative explanations for observed results | We discussed that the possibility of selection bias, misclassification bias related to exposure, and failure to consider residual or unmeasured confounding cannot be ruled out, thus, we cannot exclude chance, residual or unmeasured confounding as alternative explanation for our findings. |
| √ | Generalization of the conclusions (ie, appropriate for the data presented and within the domain of the literature review) | We discussed that the true effect would vary between the studies because of potential additional heterogeneity. Therefore, our results must be interpreted with caution. |
| √ | Guidelines for future research | We discussed that the investigators in the future study should improve the standardization of various assessment methods of head injury, dementia and AD. Furthermore, this study adds to the existing evidence that head injury may lead to neurodegenerative diseases, and the use of genetic and biological makers as surrogate end points in the future studies should help to clarify the case and effect relationship that links head injury and dementia and AD. |
| √ | Disclosure of funding source | The authors received no specific funding for this work. |
