# Supplementary material for: Head Injury as a Risk Factor for Dementia and Alzheimer’s Disease: A Systematic Review and Meta-Analysis of 32 Observational Studies
Source: PLoS One. 2017 Jan 9;12(1):e0169650. doi: 10.1371/journal.pone.0169650 (PMC5221805; doi:10.1371/journal.pone.0169650)
Supplement: S2 Table — (DOCX) [file pone.0169650.s007.docx]

**S2 Table. Quality assessment of the included studies (cohort studies)**

| Study | Selection | | | | Comparability | Outcome | | | Overall quality assessment score (of a maximum of 9) |
| --- | --- | --- | --- | --- | --- | --- | --- | --- | --- |
|  | Representativeness of the exposed cohort | Selection of the non exposed cohort | Ascertainment of exposure | Demonstration that outcome of interest was not present at start of study | Comparability of cohorts on the basis of the design or analysis | Assessment of outcome | Was follow-up long enough for outcomes to occur | Adequacy of follow up of cohorts |  |
| Abner *et al*, 2014 | * Somewhatrepresentative of the average population in the community | * Drawn from the same community as the exposed cohort | * Structured interview | * The study demonstrated that AD was not present at start of study | ** Study controls for APOE-ε4,sex,age at death,presence of at least mild cerebral amyloid angiopathy, and whether AD was observed before death | *Independent blind assessment | * The study select an adequate follow up period for outcome of interest | * Subjects lost to follow up unlikely to introduce bias (88% follow up) | 9 |
| Dams-O'Connor *et al*, 2013 | * Trulyrepresentative of the average population in the community | * Drawn from the same community as the exposed cohort | * Structured interview | * The study demonstrated that all-cause dementia was not present at start of study | * Study controls for age, age-squared, gender, and education | *Independent blind assessment | * The study select an adequate follow up period for outcome of interest | * Subjects lost to follow up unlikely to introduce bias (82% follow up) | 8 |
| Fischer *et al*, 2008 | * Somewhat representative of the average population in the community | * Drawn from the same community as the exposed cohort | * Structured interview | * The study demonstrated that dementia was not present at start of study | Study does not control for other factors | *Independent blind assessment | * The study select an adequate follow up period for outcome of interest | * Subjects lost to follow up unlikely to introduce bias (82% follow up) | 7 |
| Gardner *et al*, 2014 A | * Trulyrepresentative of the average population in the community | * Drawn from the same community as the exposed cohort | * Structured interview | * The study demonstrated that dementia was not present at start of study | ** Study controls for age, sex, race, comorbidities, trauma mechanism, health care use, and trauma severity | *Independent blind assessment | * The study select an adequate follow up period for outcome of interest | * Subjects lost to follow up unlikely to introduce bias (100% follow up) | 9 |
| Lee *et al*, 2013 | * Trulyrepresentative of the average population in the community | * Drawn from the same community as the exposed cohort | * Structured interview | * The study demonstrated that dementia was not present at start of study | ** Study controls for age, gender, urbanization level, socio-economic status, diabetes, hyperlipidemia coronary artery disease, history of alcohol intoxication, ischemic stroke, intracranial hemorrhage and Charlson comorbidity index | *Independent blind assessment | * The study select an adequate follow up period for outcome of interest | * Subjects lost to follow up unlikely to introduce bias (98% follow up) | 9 |
| Luukinen *et al*, 2005 | * Trulyrepresentative of the average population in the community | * Drawn from the same community as the exposed cohort | * Structured interview | * The study demonstrated that dementia was not present at start of study | *Study controls for low educational status and sex | *Independent blind assessment | * The study select an adequate follow up period for outcome of interest | * Subjects lost to follow up unlikely to introduce bias (82% follow up) | 8 |
| Mehta *et al*, 1999 | * Trulyrepresentative of the average population in the community | * Drawn from the same community as the exposed cohort | * Structured interview | * The study demonstrated that dementia was not present at start of study | *Study controls for age, education, and if applicable, gender | *Independent blind assessment | * The study select an adequate follow up period for outcome of interest | * Subjects lost to follow up unlikely to introduce bias (100% follow up) | 8 |
| Nordstrom *et al*, 2014 | * Trulyrepresentative of the average population in the community | * Drawn from the same community as the exposed cohort | Not reported | The study didn’t demonstrate that dementia was not present at start of study | ** Study controls for age, place and year of conscription, overall cognitive fuction, alcohol intoxication, weight, height, knee extension strength, TBI in parents, dementia in parents, income, educational level, systolic blood pressure, drug intoxication, depression, and cerebrovascular disease | *Independent blind assessment | * The study select an adequate follow up period for outcome of interest | * Subjects lost to follow up unlikely to introduce bias (100% follow up) | 7 |
| Plassman *et al*, 2000 | * Trulyrepresentative of the averagepopulation in the community | * Drawn from the same community as the exposed cohort | * Structured interview | The study didn’t demonstratethat dementia was not present at start of study | * Study controls for years of education and age | *Independent blind assessment | The study does not select an adequate follow up period for outcome of interest | * Subjects lost to follow up unlikely to introduce bias (100% follow up) | 6 |
| Schofield *et al*, 1997 | * Somewhatrepresentative of the average population in the community | * Drawn from the same community as the exposed cohort | * Structured interview | * The study demonstrated that dementia was not present at start of study | * Study controls for sex and education | *Independent blind assessment | * The study select an adequate follow up period for outcome of interest | * Subjects lost to follow up unlikely to introduce bias (100% follow up) | 8 |
| Wang *et al*, 2012 | * Trulyrepresentative of the average population in the community | * Drawn from the same community as the exposed cohort | * Structured interview | * The study demonstrated that dementia was not present at start of study | ** Study controls for sex, age group, year of index healthcare use, stroke, diabetes, hyperlipidaemia, hypertension, coronary heart disease, heart failure and atrial fibrillation | *Independent blind assessment | * The study select an adequate follow up period for outcome of interest | * Subjects lost to follow up unlikely to introduce bias (100% follow up) | 9 |
