# Supplementary material for: Head Injury as a Risk Factor for Dementia and Alzheimer’s Disease: A Systematic Review and Meta-Analysis of 32 Observational Studies
Source: PLoS One. 2017 Jan 9;12(1):e0169650. doi: 10.1371/journal.pone.0169650 (PMC5221805; doi:10.1371/journal.pone.0169650)
Supplement: S3 Table — (DOCX) [file pone.0169650.s008.docx]

**S3 Table. Quality assessment of the included studies (case-control studies)**

| Study | Selection | | | | Comparability | Exposure | | | Overall quality assessment score (of a maximum of 9) |
| --- | --- | --- | --- | --- | --- | --- | --- | --- | --- |
|  | Is the case definition adequate? | Representativeness of the cases | Selection of controls | Definition of Controls | Comparability of cases and controls on the basis of the design or analysis | Ascertainment of exposure | Same method of ascertainment for cases and controls | Non-Response rate |  |
| Bachman *et al*, 2003 | *Yes, with independent validation | * Somewhatrepresentative of the average population in the community | No description | *No history of disease | ** Study controls forage, sex, education, head trauma, alcohol, and smoking | * Secure record | * Yes | * Same rate for both groups | 8 |
| Boston *et al*, 1999 | *Yes, with independent validation | No description | *Drawn from the same community as the case | *No history of disease | ** Subjects did not show significant differences on age, social class, age of left school, family history of dementia, history of falls, history of heart attack, history of hypertension, blood pressure, smoking, drinking, psychiatric history, cholesterol, and HDL | No description | * Yes | * Same rate for both groups | 7 |
| Broe *et al*, 1990 | *Yes, with independent validation | * Somewhatrepresentative of the average population in the community | Drawn from different community as the case | *No history of disease | * Subjects were matched by sex and age | * Secure record | * Yes | * Same rate for both groups | 7 |
| Ferini-Strambi *et al*, 1990 | *Yes, with independent validation | No description | *Drawn from the same community as the case | *No history of disease | ** Subjects were matched by age, sex, residential area, education and social status | * Secure record | * Yes | * Same rate for both groups | 8 |
| Forster *et al*, 1995 | *Yes, with independent validation | * Somewhatrepresentative of the averagepopulation in the community | Drawn from different community as the case | *No history of disease | * Subjects were matched by sex and age | * Secure record | * Yes | * Same rate for both groups | 7 |
| Fratiglioni *et al*, 1993 | *Yes, with independent validation | * Somewhatrepresentative of the average population in the community | *Drawn from the same community as the case | *No history of disease | ** Study controls forage, sex, education, type of informant, and alcohol consumption | * Secure record | * Yes | * Same rate for both groups | 9 |
| Graves *et al*, 1990 | *Yes, with independent validation | * Somewhatrepresentative of the average population in the community | *Drawn from the same community as the case | *No history of disease | * Study controls for age and family history of AD | * Secure record | * Yes | * Same rate for both groups | 8 |
| Guo *et al*, 2000 | *Yes, with independent validation | * Trulyrepresentative of the average population in the community | *Drawn from the same community as the case | *No history of disease | * Study controls for gender and kinship | * Secure record | * Yes | * Same rate for both groups | 8 |
| Li *et al*, 1992 | *Yes, with independent validation | * Somewhatrepresentative of the average population in the community | *Drawn from the same community as the case | *No history of disease | * Subjects were matched by sex and age | * Secure record | * Yes | * Same rate for both groups | 8 |
| Lindsay *et al*, 2002 | *Yes, with independent validation | * Trulyrepresentative of the average population in the community | *Drawn from the same community as the case | *No history of disease | * Study controls for age, sex and education | * Secure record | * Yes | * Same rate for both groups | 8 |
| Mayeux*et al*, 1993 | *Yes, with independent validation | * Somewhatrepresentative of the average population in the community | *Drawn from the same community as the case | *No history of disease | ** Study controls for gender, age, ethnic group, years of education, and head injury | * Secure record | * Yes | * Same rate for both groups | 9 |
| McDowell *et al*, 1994 | *Yes, with independent validation | * Trulyrepresentative of the average population in the community | *Drawn from the same community as the case | *No history of disease | ** Study controls for age, sex, residence in community or institution, and education | * Secure record | * Yes | * Same rate for both groups | 9 |
| Ogunniyi *et al*, 2006 | *Yes, with independent validation | * Somewhatrepresentative of the average population in the community | *Drawn from the same community as the case | *No history of disease | * Study controls for age and gender | * Secure record | * Yes | * Same rate for both groups | 8 |
| O'Meara *et al*, 1997 | *Yes, with independent validation | * Trulyrepresentative of the average population in the community | *Drawn from the same community as the case | *No history of disease | Study does not control for other factors | * Secure record | * Yes | * Same rate for both groups | 7 |
| Rasmusson *et al*, 1995 | No, the case definition was not adequate | * Somewhatrepresentative of the average population in the community | Drawn from different community as the case | *No history of disease | Study does not control for other factors | * Secure record | * Yes | * Same rate for both groups | 5 |
| Rippon *et al*, 2006 | *Yes, with independent validation | * Somewhatrepresentative of the average population in the community | *Drawn from the same community as the case | *No history of disease | * Study controls forε4 status, age, gender, and education | * Secure record | * Yes | * Same rate for both groups | 8 |
| Salib *et al*, 1997 | *Yes, with independent validation | * Somewhatrepresentative of the average population in the community | *Drawn from the same community as the case | *No history of disease | ** Study controls for age, sex, time lag between head injury and onset, duration of condition and family history of dementia | * Secure record | * Yes | * Same rate for both groups | 9 |
| Suhanov*et al*, 2006 | *Yes, with independent validation | * Somewhatrepresentative of the average population in the community | *Drawn from the same community as the case | *No history of disease | * Study controls for family history of dementia, family history of parkinsonism, and hypertension | * Secure record | * Yes | * Same rate for both groups | 8 |
| Sundstrom *et al*, 2007 | *Yes, with independent validation | * Somewhatrepresentative of the average population in the community | *Drawn from the same community as the case | *No history of disease | * Subjects were matched by sex and age | * Secure record | * Yes | * Same rate for both groups | 8 |
| Tsolaki *et al*, 1997 | *Yes, with independent validation | * Somewhatrepresentative of the average population in the community | *Drawn from the same community as the case | *No history of disease | * Subjects were matched by sex and age | * Secure record | * Yes | * Same rate for both groups | 8 |
| vanDuijn *et al*, 1992 | *Yes, with independent validation | * Trulyrepresentative of the average population in the community | *Drawn from the same community as the case | *No history of disease | * Study controls for age, sex, dementia in first-degree relatives and education | * Secure record | * Yes | * Same rate for both groups | 8 |
